# Advanced microRNA-based cancer diagnostics using amplified time-gated FRET†
†Electronic supplementary information (ESI) available. See DOI: 10.1039/c8sc03121e


**DOI:** 10.1039/c8sc03121e

**Published:** 2018-09-11

**Authors:** Xue Qiu, Jingyue Xu, Jiajia Guo, Akram Yahia-Ammar, Nikiforos-Ioannis Kapetanakis, Isabelle Duroux-Richard, Julia J. Unterluggauer, Nicole Golob-Schwarzl, Christophe Regeard, Catherine Uzan, Sébastien Gouy, Michael DuBow, Johannes Haybaeck, Florence Apparailly, Pierre Busson, Niko Hildebrandt

**Affiliations:** a NanoBioPhotonics , Institute for Integrative Biology of the Cell (I2BC) , Université Paris-Saclay , Université Paris-Sud , CNRS , CEA , Orsay , France . Email: niko.hildebrandt@u-psud.fr ; https://www.nanofret.com; b Gustave Roussy , Université Paris-Saclay , CNRS , UMR 8126 , Villejuif , France; c Université Paris-Sud , Université Paris-Saclay , Le Kremlin-Bicêtre , France; d IRMB , INSERM , Univ Montpellier , Montpellier , France; e Diagnostic and Research Institute of Pathology , Diagnostic and Research Center for Molecular BioMedicine , Medical University of Graz , Austria; f Laboratoire de Génomique et Biodiversité Microbienne des Biofilms (LGBMB) , Institute for Integrative Biology of the Cell (I2BC) , Université Paris-Saclay , Université Paris-Sud , CNRS , CEA , Orsay , France; g Department of Surgery , Gustave Roussy , Université Paris-Saclay , Villejuif , France; h Department of Breast and Gynecologic Surgery , Pitié Salpêtrière Hospital , APHP , Institut Universitaire de Cancérologie , Sorbonne University , INSERM U938 , France; i Department of Pathology , Otto-von-Guericke-University Magdeburg , Germany; j Department of Pathology , Medical University Innsbruck , Austria; k Clinical Department for Osteoarticular Diseases , University Hospital of Montpellier , Montpellier , France

## Abstract

MicroRNAs (miRNAs) play an important role in cellular functions and in the development and progression of cancer. Precise quantification of endogenous miRNAs from different clinical patient and control samples combined with a one-to-one comparison to standard technologies is a challenging but necessary endeavor that is largely neglected by many emerging fluorescence technologies. Here, we present a simple, precise, sensitive, and specific ratiometric assay for absolute quantification of miRNAs. Isothermally amplified time-gated Förster resonance energy transfer (TG-FRET) between Tb donors and dye acceptors resulted in miRNA assays with single-nucleotide variant specificity and detection limits down to 4.2 ± 0.5 attomoles. Quantification of miR-21 from human tissues and plasma samples revealed the relevance for breast and ovarian cancer diagnostics. Analysis of miR-132 and miR-146a from acute monocytic leukemia cells (THP-1) demonstrated the broad applicability to different miRNAs and other types of clinical samples. Direct comparison to the gold standard RT-qPCR showed advantages of amplified TG-FRET concerning precision and specificity when quantifying low concentrations of miRNAs as required for diagnostic applications. Our results demonstrate that a careful implementation of rolling circle amplification and TG-FRET into one straightforward nucleic acid detection method can significantly advance the possibilities of miRNA-based cancer diagnostics and research.

## Abstract 2

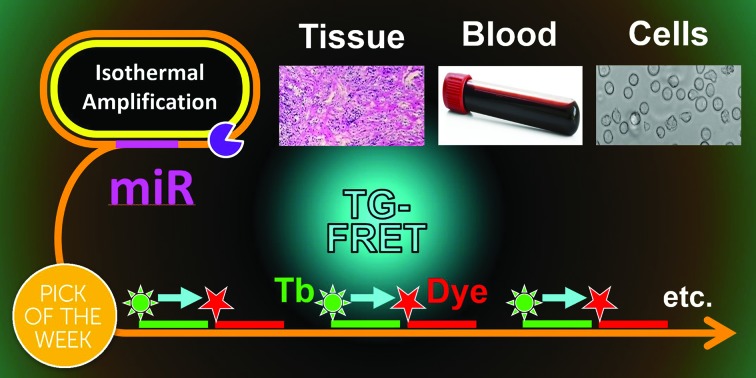
FRET and rolling circle amplification outperform RT-qPCR for microRNA diagnostics in clinical samples.

## Introduction

MicroRNA-based diagnostics has the potential to play a pivotal role for the prognosis, diagnosis, management, and monitoring of diseases because varying expression levels can be detected in most solid (cells and tissues) and liquid (*e.g.*, blood, urine, saliva) clinical samples.^1^–^5^ Despite the rapid growth of miRNA research over the last decade and the relation of many different miRNAs to various pathologies, miRNA has remained a next-generation biomarker and the translation into routine clinical practice is progressing slowly. Although the importance and potential of miRNA-diagnostics have been largely acknowledged, challenges related to, *e.g.*, different expression levels in solid and liquid biopsies, technological limitations, lacking disease-specific miRNA signatures, workflow standardization, and sample properties and preparation methods, must be overcome.^1^,^2^,^4^,^5^

Of the standard miRNA detection methods, such as reverse transcription quantitative PCR (RT-qPCR), next generation sequencing (NGS), or microarrays, RT-qPCR is arguably the most suited technology for routine and simple use.^1^,^2^ However, the necessity of reverse transcription and multiple primers (for RT and qPCR), the sensitivity to contamination generated by RNA extraction, and the rather extensive guidelines for obtaining reliable results with qPCR present serious drawbacks for simple and reliable analysis.^6^ The large majority of clinical studies that have related different miRNAs in solid (tissues) and liquid (blood) biopsies to various cancers, infectious and cardiac diseases, diabetes, and sepsis, have used relative concentrations because absolute quantitation is impossible with NGS and too imprecise with RT-qPCR.^3^,^5^,^7^–^14^ On the contrary, highly selective, sensitive, and precise absolute quantitation, as commonly applied in immunoassays,^15^ is probably one of the most important aspects for successfully translating the use of miRNA biomarkers to the clinic.

Novel methods that can overcome the technical limitations of the standard technologies,^4^,^5^,^16^ while at the same time providing the necessary sensitivity to quantify miRNAs in clinical samples, have a strong potential to significantly advance translational research. Many emerging technologies have been developed over the recent years and the most frequently applied strategies include nanoparticle-based fluorescence detection and DNA amplification with optical or electrochemical readout.^17^–^23^ Target-primed rolling circle amplification (RCA) combined with different luminescence readout strategies can be used for specific and sensitive detection of miRNAs.^17^,^24^–^27^ Based on previous work concerning DNA detection with combined RCA and FRET from a dye to a quencher by Zhou *et al.*,^28^ Wu *et al.* developed an RCA-FRET miRNA assay with steady-state detection of two fluorescent dyes as FRET pair.^26^ Although this proof of concept study could provide a very low detection limit of 103 aM and reliable detection above background of 6 fM, important requirements for an applicable miRNA assay, such as probe versatility, precision, reproducibility, comparison to a standard technology, and the detection of endogenous miRNAs in real clinical samples, were missing.^26^ Due to missing proofs for relevant clinical application, none of the emerging fluorescence-based technologies proposed for simple miRNA detection has been translated into routine clinical practice.

**Fig. 1:**
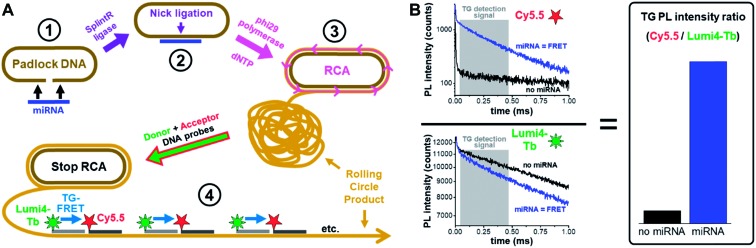
Principle of miRNA detection by amplified TG-FRET. (A) After specific recognition of miRNA by a linear padlock DNA (1), the DNA padlock nick is ligated over the miRNA target splint using SplintR ligase (2) and the miRNA becomes a primer for a phi29 polymerase to synthesize and displace (by RCA) complimentary DNA around the circularized padlock DNA (3). After stopping RCA, the rolling circle product (RCP) is incubated with Tb (Lumi4-Tb) donor and Cy5.5 acceptor labeled ssDNA, which hybridize to specific sequences that exist more than 1000-fold on the amplified RCP concatemer. The close distance of Lumi4-Tb and Cy5.5 in the RCP allows for Tb-to-Cy5.5 FRET, which is not possible if both are free in solution (not hybridized to the RCP). Thus, the TG-FRET signal can be used for quantifying miRNA without any washing or separation steps. (B) Ratiometric TG-FRET, which measures the ratio of FRET-sensitized Cy5.5 photoluminescence (PL) and FRET-quenched Tb PL within a specific time-window after pulsed excitation (to suppress autofluorescence), is used to quantify the miRNA target in a 140 μl microwell within 5 seconds.

To advance this translational research endeavor, we implemented the ratiometric and single-step detection format of time-gated FRET (TG-FRET)^29^,^30^ from a Tb donor to a Cy5.5 acceptor in target-primed RCA.^31^ Amplified TG-FRET follows a straightforward sample-preparation workflow (Fig. 1A), and TG-FRET detection (Fig. 1B) only takes a few seconds on a commercially available clinical immunofluorescence plate reader (KRYPTOR). Application of SplintR ligase allowed for efficient ligation of the DNA padlock probe over the miRNA target and could therefore detect miRNA at very low concentrations without prior reverse transcription of miRNA to cDNA. In contrast to many other emerging technologies, amplified TG-FRET can not only detect very low amounts of miRNAs, but also provides extremely high specificity against precursor miRNA (pre-miRNA) and miRNAs with single nucleotide variations. We demonstrate the immediate practical applicability by precise quantification of endogenous miRNAs from various types of clinical samples and for different pathologies, namely miR-21 from human plasma and tissue related to ovarian and breast cancer^32^,^33^ and miR-132 and miR-146a from *in vitro* cultured cells (THP-1) related to innate immune responses.^34^ At the low miRNA concentrations required for clinical diagnostics, amplified TG-FRET provided both better specificity (distinction of single-nucleotide variations at varying positions within the target) and higher precision (smaller distribution of concentrations and better distinction between healthy and pathological samples) than the gold standard method RT-qPCR.

## Results and discussion

### Sensitivity and dynamic range

To quantify miRNA, amplified TG-FRET technology applies time-gated (0.1–0.9 ms after pulsed excitation) ratiometric PL intensity (FRET-ratio) detection (Fig. 1B) of Cy5.5 acceptors and Lumi4-Tb (Tb) donors (*cf.* ESI Fig. S1† for absorption/emission spectra and FRET parameters), which only takes a few seconds on a KRYPTOR immunofluorescence plate reader. We first evaluated the performance for miRNA quantification on exogenous miR-21, miR-132, and miR-146a. SplintR ligase was used for efficient ligation of the DNA padlock probes over the miRNA targets. Although shorter padlock probes were shown to exhibit higher fluorescence intensities in RCA-FRET,^26^ we chose padlock probes of 77 and 78 nucleotides (see Table 1 for sequences of all RNAs and DNAs used in the study). These relatively long DNAs allowed us to avoid overlap between the sequences that recognized the miRNA targets and those that hybridized the Tb-donor and dye-acceptor DNA probes and to optimize the FRET distance between Tb and dye inside the RCA product. The actual Tb-dye distance in the rolling circle product (RCP, Fig. 1A) was 18 base pairs (see Table 1 for complementary sequences between the padlock and the Tb and dye probes). This distance would correspond to *ca.* 7 nm when using 0.34 nm per base pair and 0.4 ± 0.1 nm for both Tb and Cy5.5 and was shown to be in excellent agreement with time-resolved FRET measurements on double-stranded (ds) DNA.^35^ When comparing the PL decays of the Tb-Cy5.5 FRET pair inside the RCP and inside dsDNA (ESI Fig. S2†), the average FRET-sensitized PL lifetime is significantly shorter for the RCP FRET-pair (0.4 ms *vs.* 2 ms), which means that the average FRET efficiency is higher (0.86 *vs.* 0.28). This difference can be explained by the folded (coiled) structure of the RCP and a concomitant closer donor–acceptor distance and/or the interaction of one donor with several acceptors, both of which lead to higher FRET efficiencies. Despite the different behavior of the Tb-dye FRET pair in a folded RCP concatemer, donor–acceptor distance adjustment (different number of base pairs between Tb and dye) can still be used to produce distinct PL decays that are applicable for multiplexed nucleic acid detection.^31^ Another practical reason of the non-overlapping sequences for miRNA targets and fluorescent probes was the usability of the same Tb-donor and dye-acceptor probes for different miRNA targets (see color code in Table 1). Taking into account the many different miRNAs that have been related to various cancers,^36^,^37^ this versatile padlock design is an important advantage for diagnostic applications.

**Table 1:** Sequences and modifications of all DNA and RNA probes and targets. Sequence differences shown in red, sequence similarities shown in magenta, target-specific termini of padlock DNA shown in blue, Tb-probe-complementary sequences shown in green, and Cy5.5-probe-complementary sequences shown in orange

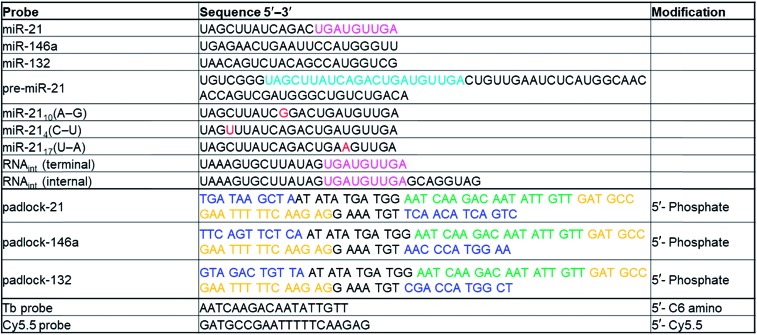

**Fig. 2:**
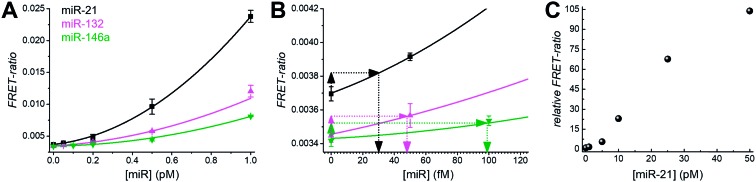
(A) Calibration curves for miR-21, miR-132, and miR-146a for concentrations between 0.05 and 1.0 pM within 140 μl solutions per microwell. (B) Enlarged view of the same calibration curves in the 0 to 120 fM concentration range. LODs were determined as shown (using 3 standard deviations of the blank) to 30 ± 3 fM for miR-21, 48 ± 5 fM for miR-132, and 99 ± 10 fM for miR-146a. (C) Assay calibration curve of miR-21 over a larger concentration range (up to 50 pM) to assess the dynamic range of amplified TG-FRET assays.

For all three miRNA targets the calibration curves (Fig. 2A) showed increasing FRET-ratio values with increasing miRNA concentrations. Limits of detection (LODs) were determined (three standard deviations over the blank sample) as 4.2 ± 0.5 attomole (30 ± 3 fM) for miR-21, 6.8 ± 0.8 attomole (48 ± 5 fM) for miR-132, and 14 ± 2 attomole (99 ± 10 fM) for miR-146a (Fig. 2B) and the dynamic concentration range spanned more than 3 orders of magnitude (∼50 fM to ∼50 pM, Fig. 2C).

### Specificity and comparison to RT-qPCR

**Fig. 3:**
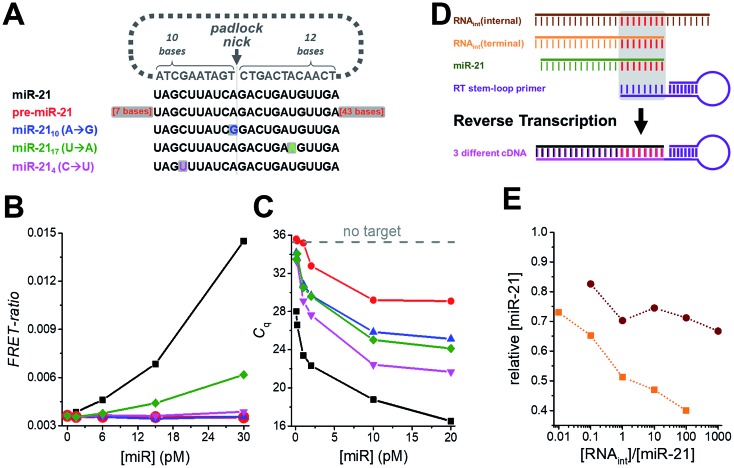
(A) The specificity of amplified TG-FRET and TaqMan RT-qPCR for miR-21 (black) was challenged against pre-miR-21 (red) and three different targets with single nucleotide variations compared to miR-21 (blue, green, and magenta). (B) For amplified TG-FRET, only the mismatch at a remote position from the padlock nick on the longer (12 nt) target-recognizing terminus led to a nonspecific signal at elevated concentrations of miRNA (miR-21_17_ (U → A), green curve). The versatile probe design can overcome this problem by using a padlock probe with a nick closer to the mismatch (miR-21_10_ (A → G), blue curve) or with the shorter target-recognizing terminus (10 nt) over the mismatch (miR-21_4_ (C → U), magenta curve). Pre-miR-21 (red curve) did not lead to any nonspecific signal either. The black curve shows the signal for the miR-21 target (without any mismatches). (C) TaqMan RT-qPCR is strongly influenced by pre-miR-21 (red curve) and all three single nucleotide variations (blue, green, and magenta curve), as shown by decreasing quantification cycles (*C*_q_) with increasing mismatch concentrations (within the same low picomolar miRNA concentration range as for TG-FRET – *cf.* graph in B). Gray dashed line indicates *C*_q_ value for samples without target. (D) The short target-hybridization sequence of the TaqMan RT-stem loop primer can lead to reverse transcription of RNA that have the same internal or terminal sequence than the target terminus. (E) Both internal and terminal interfering sequences led to negative offsets (down to 40% for RNA_int_(terminal)) of the target concentration ([miR-21] = 10 pM).

Another important requirement for diagnostics is specificity at very low concentrations, which we verified by challenging the miR-21 assay with various amounts of precursor miR-21 (pre-miR-21) as well as three modified miR-21 targets with single nucleotide variations at different positions (Fig. 3A). Compared to samples without target, pre-miR-21 did not lead to any signal increase for TG-FRET (red curve in Fig. 3B) but significantly reduced quantification cycles (*C*_q_) for RT-qPCR (red curve in Fig. 3C), which will lead to an overestimation (positive offset) of the real miR-21 target concentration (the lower the *C*_q_ the higher the measured concentration). Although the *C*_q_ values for the miR-21 target were much lower (black curve in Fig. 3C), the nonspecific pre-miR-21 signal suggests that the internal miR-21 sequence of pre-miR-21 (with additional 7 bases on the 5′ end and 43 bases on the 3′ end) can still be reverse transcribed and amplified by RT-qPCR albeit with much lower efficiency compared to miR-21. For TG-FRET, it is very likely that the miR-21 sequence inside pre-miR-21 will be specifically hybridized by the padlock DNA. However, the two 3′ and 5′ overhangs prevent amplification by the phi29 polymerase. Although it was shown that phi29 polymerase can provide 3′ → 5′ RNA exonucleolytic activity, the efficiency was ∼10-fold lower than for DNA.^38^ We therefore assume that, under our experimental conditions, the additional 43 bases on the 3′ end cannot be hydrolyzed efficiently enough to initiate RCA. Concerning the single-nucleotide variations of miR-21, only miR-21_17_ (U → A) at concentrations higher than 6 pM led to a significant miR-21 signal for TG-FRET (green curve in Fig. 3B). This nonspecific signal resulted from a single-nucleotide variation at a distant position from the padlock nick on the longer (12 nt) target-recognizing terminus of the padlock DNA (Fig. 3A). The versatility of the padlock design can overcome even such particular mismatches. Moving the nick closer to the mismatch and/or shortening the length of the mismatch-recognizing terminus (10 nt) completely removed the non-specific signal as shown by two other mismatched miR-21 variants (miR-21_10_ (A → G) and miR-21_4_ (C → U) – blue and magenta curves in Fig. 3B). In contrast, the gold standard RT-qPCR was strongly influenced by all three mismatched miR-21 (Fig. 3C). Although the quantification cycles (*C*_q_) were distinguishable from the original miR-21 target, they significantly decreased to values far below that seen without any target. While such mismatches will result in a positive offset of the real miR-21 target concentration, the RT step of RT-qPCR can lead to negative concentration offsets. The presence of internal or terminal RNA sequences that are complementary to the very short hybridization sequence (∼6 to 8 nt) of the RT stem-loop primer may be recognized by the RT primer and therefore reduce RT efficiency and produce less cDNA (Fig. 3D). Indeed, both internal and terminal complementary sequences of otherwise unrelated RNAs led to a significant decrease in the measured miR-21 concentrations (Fig. 3E). Such short sequences that are complementary to the RT stem-loop primer may exist in many different RNAs present in real clinical samples and present a serious drawback for RT-qPCR-based diagnostics. These results clearly show another important advantage of amplified TG-FRET when it comes to clinical applications.

### miRNA detection in human plasma, tissue, and cells

To demonstrate immediate applicability of amplified TG-FRET to biologically relevant samples and research, we quantified hsa-miR-21 in 26 plasma samples related to ovarian cancer (13 samples from ovarian cancer patients and 13 samples from healthy individuals), hsa-miR-21 in 10 tissue samples related to breast cancer (4 healthy tissues and 6 primary tumor tissues), and hsa-miR-132 and hsa-miR-146a in lysate samples from 0.25 × 10^6^ and 3 × 10^6^ THP-1 cells stimulated with lipopolysaccharide (LPS) for 0 h or 24 h. For comparison, all samples were also quantified with TaqMan RT-qPCR.

**Fig. 4:**
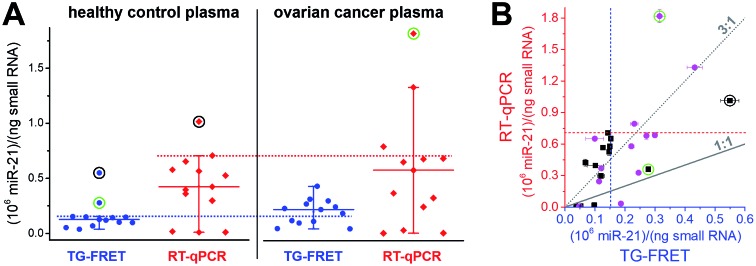
(A) Absolute quantification of hsa-miR-21 in human plasma (ovarian cancer) using amplified TG-FRET (blue) and RT-qPCR (red). miR-21 concentrations (in small-RNA extracts) of 13 healthy control and 13 ovarian cancer samples were quantified. Circles around data points indicate statistical outliers (in green for only one of the techniques, in black for both techniques). Whiskers represent maximum and minimum values (without outliers) and horizontal lines represents the median. Dotted lines represent a threshold value (maximum of healthy control samples). More information about samples and human research participants can be found in ESI Tables S1 and S2.† All concentrations are given in copy number per ng of small RNA. Absolute (molar) concentrations are shown in ESI Fig. S3.† (B) Scatterplot comparing RT-qPCR with TG-FRET concentrations for the same samples. Healthy samples are shown in black and cancer samples in magenta. For orientation, lines of perfect agreement (1 : 1) between the two techniques and 3-fold higher RT-qPCR values (3 : 1) are shown. Circles around data points indicate the statistical outliers from (A). Blue and red dashed lines present the threshold values from (A).

Plasma samples are arguably the most challenging for miRNA-based diagnostics because of the low miRNA concentrations and the large variations in miRNA expression. As shown in Fig. 4A, amplified TG-FRET values provided a much narrower distribution of concentrations compared to RT-qPCR. When applying a threshold value (maximum concentration in healthy samples without outliers), TG-FRET could reveal a significant difference between healthy and ovarian cancer samples (62% above and 38% below threshold line), whereas RT-qPCR showed only minor differences (17% above and 83% below threshold line). Comparing all samples in a scatter plot (TG-FRET *vs.* RT-qPCR concentrations, Fig. 4B) showed the difference between both techniques. Although samples were distributed below and above the ideal 1 : 1 line, a trend toward higher RT-qPCR concentrations (*circa* 3 : 1) became evident. The scatter plot also showed a large concentration distribution of both healthy and cancer samples and the better distinction of cancer and healthy samples for TG-FRET. Receiver operating characteristic (ROC) curves (ESI Fig. S4†) indicated that both TG-FRET and RT-qPCR provided better diagnostic performance than a random guess but that TG-FRET can significantly better classify healthy and cancer samples. Although the results showed a better analytical performance of TG-FRET, they also confirmed the challenges of miRNA quantification in plasma and that larger sample cohorts are necessary to provide a clear clinical conclusion.^39^ Nevertheless, the outcome of this study on 26 different plasma samples clearly showed that amplified TG-FRET has a large potential to add significant advantages to clinical studies with large patient cohorts.

**Fig. 5:**
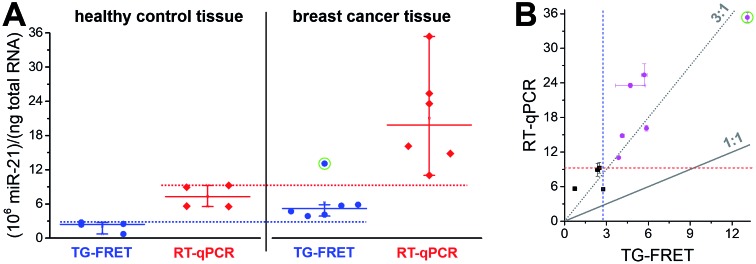
(A) Absolute quantification of hsa-miR-21 in human tissue (breast cancer) using amplified TG-FRET (blue) and RT-qPCR (red). miR-21 concentrations (in total-RNA extracts) of four healthy tissues (non-neoplastic breast) and six breast tumor tissues were quantified. Circle around data point indicates statistical outlier. Whiskers represent maximum and minimum values (without outlier) and horizontal lines represents the median. Dotted lines represent a threshold value (maximum of healthy control samples). More information about samples and human research participants can be found in ESI Tables S1 and S2.† All concentrations are given in copy number per ng of total RNA. Absolute (molar) concentrations are shown in ESI Fig. S3.† (B) Scatterplot comparing RT-qPCR with TG-FRET concentrations for the same samples. Healthy samples are shown in black and cancer samples in magenta. For orientation, lines of perfect agreement (1 : 1) between the two techniques and 3-fold higher RT-qPCR values (3 : 1) are shown. Circle around data point indicates the statistical outlier from (A). Blue and red dashed lines present threshold values from (A).

A narrower distribution of TG-FRET-determined concentrations was also found for breast cancer tissues (Fig. 5A). Although we disposed fewer samples compared to plasma, a distinction of healthy from cancer samples was evident. One of the reasons may be the higher absolute concentrations (pM in plasma and nM in tissue – *cf.* ESI Fig. S3a and b†) because RT-qPCR is known to be less precise at low target input.^16^ Both amplified TG-FRET and RT-qPCR could very well distinguish healthy and cancer tissue (100% of healthy samples below threshold and 100% of cancer samples above threshold). Similar to the plasma results, miR-21 concentration values for RT-qPCR were significantly higher (also approximately 3-fold) compared to TG-FRET but there was much less distribution in the scatterplot when comparing both techniques (Fig. 5B). Although the patient cohort was relatively small, the results clearly show that amplified TG-FRET can provide very useful clinical information (healthy or cancer) at a relatively simple workflow without reverse transcription and without interferences from reagents used in RNA extraction. TG-FRET may therefore be used as a stand-alone or complementary analytical method to accomplish higher precision in miRNA-based tissue diagnostics.

**Fig. 6:**
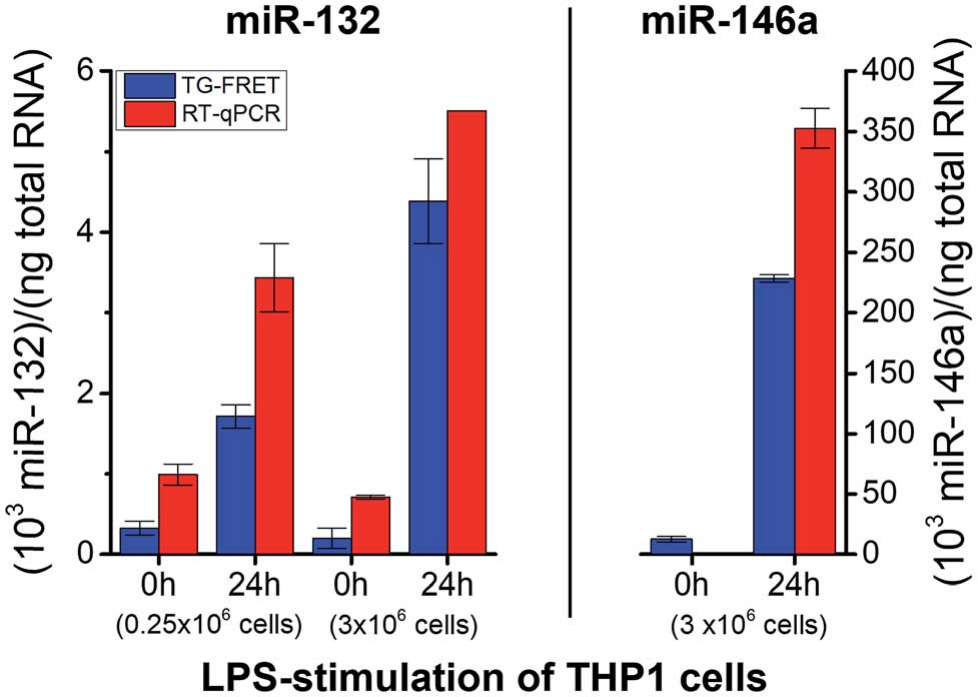
Absolute quantification of hsa-miR-132 and hsa-miR-146a in *in vitro* cultured cells (LPS stimulation) using amplified TG-FRET (blue) and RT-qPCR (red). miR-132 (left) and miR-146a (right) concentrations (in total-RNA extracts) of THP-1 cells after 0 h and 24 h of stimulation with LPS (1 μg ml^–1^). Note: value of miR-146a for 0 h stimulation measured with RT-qPCR is 0.9 and therefore not visible within the concentration scale. More information about the samples can be found in ESI Table S1.† All concentrations are given in copy number per ng of total RNA. Absolute (molar) concentrations are shown in ESI Fig. S3.†

To demonstrate successful application of amplified TG-FRET beyond cancer diagnostics and for a third sample type, quantification of two other endogenous miRNAs (hsa-miR-132 and hsa-miR-146a) was performed in total-RNA extracts from LPS-stimulated THP-1 cells (Fig. 6). LPS stimulation resulted in a significant increase in miR-132 concentrations for both smaller (250 000 cells) and larger (3 × 10^6^ cells) amounts of cells. The higher amount of cells allowed us to produce more total-RNA but at approximately equal concentrations (*cf.* ESI Table S1†). Similar to plasma and tissue measurements, concentration values were (2.4 ± 1.1)-fold higher for RT-qPCR compared to TG-FRET. These higher concentration values in all three types of clinical samples suggest that the positive concentration offset of RT-qPCR (*cf.*Fig. 3A–C) caused by miRs with very similar sequences is significantly stronger than the negative concentration offset (*cf.*Fig. 3E) caused by the RT step of RT-qPCR. Absolute concentrations (*cf.* ESI Fig. S3†) were similar to plasma samples (pM concentration range). Average relative increases due to LPS stimulation were 5.3/22-fold and 3.5/7.8-fold (smaller/larger amounts of cells) for TG-FRET and RT-qPCR, respectively. The larger amount of cells was used for the quantification of miR-146a. Concentration values were 14-fold lower (no stimulation) and 1.5-fold higher (24 h stimulation) for RT-qPCR, and this time the relative increases due to LPS stimulation were 18-fold and 395-fold for TG-FRET and RT-qPCR, respectively. TG-FRET showed better agreement with a previous study, which reported that both miR-132 and miR-146a showed equivalent elevation and that the one of miR-146a was circa 8-fold (normalized to 5S RNA).^34^ Again, we used only few samples to demonstrate quantification of miRNAs in cells and though the results were more consistent for TG-FRET, a larger study would be necessary to confirm its better performance. However, similar to the tissue samples, we could clearly show (and verify by RT-qPCR) that TG-FRET can efficiently distinguish LPS-stimulated from non-stimulated cells and that this new technology has the potential to become a useful tool for cell-based miRNA diagnostics.

## Conclusions

The importance of miRNAs in biological regulation, cell function, and the development and progression of cancer and other diseases^3^,^7^,^9^,^12^,^13^ in combination with challenging biological and analytical conditions^4^,^5^,^16^ have led to the emergence of many miRNA detection technologies, all with specific advantages and drawbacks.^1^,^39^–^46^ Highly important requirements for a new technology to become a useful complementary miRNA detection method and to advance miRNA-based diagnostics and translational research are not only sensitivity and specificity but also the capability to be applicable to different types of clinical samples and to obtain useful clinical information from those samples.

In our study, we have presented the development, characterization, and application of a new amplified TG-FRET technology for absolute quantification of miRNA. Amplified TG-FRET uses simple isothermal RCA and ratiometric TG-FRET detection for precise quantification on a commercial clinical plate reader (KRYPTOR). The rapid miRNA assay does not require any separation or washing steps and can directly quantify miRNA without reverse transcription to cDNA. To fulfill the technological and clinical requirements we designed TG-FRET for high sensitivity, specificity, and the use for quantifying miRNA from human plasma, tissues, and cells. Careful characterization of sensitivity and specificity for miR-21, miR-132, and miR-146a revealed LODs down to 4.2 attomoles (30 fM), efficient distinction against single-nucleotide variations and precursor miRNA, and significant advantages in specificity compared to RT-qPCR.

To evaluate the immediate applicability to a broad range of biological or clinical studies, we tested the assay performance on small groups of different types of samples (plasma, tissue, and cells) with different pathological backgrounds (ovarian cancer, breast cancer, and innate immune response) in a one-to-one comparison with TaqMan RT-qPCR. While hsa-miR-21 quantification from tissue allowed for a clear distinction between healthy and tumor tissue and hsa-miR-132 and hsa-miR-146a quantification from THP-1 cells provided clear evidence for LPS-stimulated or non-stimulated cells, pathological evaluation of plasma samples appeared more challenging. From the diagnostic point of view, TG-FRET clearly outperformed RT-qPCR with 62% of cancer plasma samples detected above the maximum concentration value of healthy control samples, whereas RT-qPCR only detected 17% above threshold. For most of the samples (independent of the sample type), RT-qPCR detected higher concentrations (approximately 3-fold) than TG-FRET and RT-qPCR determined concentrations with a significantly larger distribution. We attribute these higher and broader values of RT-qPCR to specificity issues (*cf.*Fig. 3), the susceptibility to interferences with sample extraction reagents, and the lower precision of this exponential amplification technique at low target input. The ability to quantify different miRNAs from various types of clinically relevant samples, to obtain significant pathological information from these measurements, the advantages in simplicity and precision compared to RT-qPCR, and the adaptability of the isothermal and washing-free procedure to life-cell imaging, will make TG-FRET a very useful tool (in combination with other technologies or as stand-alone method) for advancing miRNA-based diagnostics and research. Moreover, amplified TG-FRET has the potential to create significant impact for the translation of miRNA biomarkers into the clinic.

## Materials and methods

### Nucleic acid probes and exogenous targets

All sequences and modifications of nucleic acids are summarized in Table 1. All oligonucleotides were purchased from Eurogentec. Phosphate DNAs were purified with polyacrylamide gel electrophoresis. All other modified DNAs and RNAs were purified with HPLC. Lumi4-Tb-NHS (Tb-NHS) was provided by Lumiphore, Inc. Tb-DNA conjugation was performed as described elsewhere.^18^ Briefly, Tb-DNA conjugates were obtained by mixing Tb-NHS in concentration excess to amino-functionalized oligonucleotides in 100 mM carbonate buffer at pH 9.0. The mixtures were incubated overnight at 4 °C. The Tb-DNA conjugates were purified 3 times with HEPES buffer (100 mM, pH 7.4) by Zeba Spin Desalting Columns (7 kDa MWCO). Tb concentrations were determined by absorbance measurements at 340 nm using a molar absorptivity of 26 000 M^–1^ cm^–1^ as provided by the manufacturer. DNA was quantified by absorbance measurements at 260 nm. Conjugation ratios were determined by linear combination of the respective absorbance values of Tb and oligo within the Tb-oligo conjugates and were in all cases higher than 0.9 Tb/DNA.

### Photophysical analysis

Absorption spectra (Lambda 35 UV/Vis System, PerkinElmer) and emission spectra (FluoTime 300, PicoQuant) were recorded in HEPES buffer (100 mM, pH 7.4) and deionized water (purified by Purelab Option-Q equipped with bio-filter, ELGA LabWater) for Tb and Cy5.5 samples, respectively.

### Amplified TG-FRET miRNA assays

miRNA assays were prepared on a clean bench. In a typical assay, 1.5 nM padlock probe and an appropriate amount of the target miRNA were prepared in 10 μl optimized SplintR DNA ligase reaction buffer (BUFFER-1, 50 mM Tris–HCl, 10 mM MgCl_2_, 100 mM NaCl, 10 μM ATP, 10 mM DTT, pH 7.6), and the mixture was incubated in a thermal cycler with a temperature control program (80 °C for 2 min → decreased from 80 °C to 22 °C with a 2°C min^–1^ speed). Then, 21.5 U of SplintR DNA ligase (New England Biolabs) prepared in 5 μl BUFFER-1 was added to the mixture and incubated at 37 °C for 1 h. Afterwards, 15 μl phi29 DNA polymerase reaction buffer (BUFFER-2, 1× buffer components: 50 mM Tris-Cl, 10 mM MgCl2, 10 mM (NH_4_)_2_SO_4_, 4 mM DTT, pH 7.4), which contained 5 U of phi29 DNA polymerase (New England Biolabs) and 0.5 mM dNTP (New England Biolabs), was added and incubated at 37 °C for 3 h. Before termination of the polymerization process, 2.5 nM Tb probe and 2.5 nM Cy5.5 probe prepared in 120 μl hybridization buffer (BUFFER-3, 20 mM Tris-Cl, 500 mM NaCl, 0.1% BSA, pH 8.0) were added and then incubated in a thermal cycler with a temperature control program (65 °C for 10 min → decreased from 65 °C to 22 °C with a 2°C min^–1^ speed → 22 °C for 10 min). From the total reaction volume of 150 μl, 140 μl were measured in black 96-well microtiter plates on the clinical immunofluorescence plate reader KRYPTOR compact plus (Thermo Fisher Scientific) with time-gated (0.1–0.9 ms) PL intensity detection using optical bandpass filters (Semrock) with 494 ± 12 nm for the Tb detection channel and 716 ± 20 nm for the Cy5.5 detection channel. For ratiometric analysis, FRET-ratios were calculated by the ratio of Cy5.5 and Tb time-gated PL intensities.

### RNA extraction

Tissue material was obtained with informed consent at the Medical University of Graz and the St. John of God Hospital Graz under approval from the ethics committee of the Medical University of Graz and the ethics committee of the St. John of God Hospital Graz (23-015 ex 10/11). All breast tissue samples were gathered in course of routine interventions and autopsies, respectively, and used in an anonymized/pseudonymized way. The use was approved by the responsible ethics committee. All plasma samples were collected from anonymized patients with written informed consent under the agreement of the responsible ethical committees (official permission *n*° 2746 by the “comité de protection des personnes Ile de France III” – January 5, 2010). For all plasma samples, miRCURY™ RNA Isolation Kit -Biofluids (Exiqon) was used for the extraction of small RNA. The extraction was performed according to the manufacturer's instructions. In a typical experiment, 250 μl of each plasma sample was centrifuged at 3000*g* for 5 min to pellet any debris and insoluble components after thawing. 200 μl of supernatant was transferred to a new tube and 60 μl Lysis solution and 1.2 μl MS2 RNA (Sigma-Aldrich) were added into the solution for 3 min at room temperature to lyse plasma components. After that, 20 μl protein precipitation solution was added for 1 min at room temperature. The precipitated mixture was centrifuged for 3 min at 11 000*g* and the clear supernatant was transferred into a new collection tube. Then, 270 μl isopropanol was added to the collected supernatant, and the solution was loaded to a microRNA Mini Spin Column for 2 min at room temperature. After centrifuging for 30 s at 11 000*g*, the solution was washed once with 100 μl washing solution 1 and twice with washing solution 2 (700 μl and 250 μl). Finally, the RNA was eluted with 50 μl RNase free water, and all RNA extractions were stored at –80 °C. Total RNA was isolated from snap-frozen human breast tissue samples using Trizol reagent (Life Technologies), followed by extraction with phenol-chloroform. THP-1 cells were grown in RPMI 1640 supplemented with 10% fetal calf serum, 1% penicillin/streptomycin, and l-glutamine. THP-1 cells were not treated (0 h) or treated for 24 h (24 h) with LPS (1 μg ml^–1^). Total RNAs were extracted using a miRNeasy Mini Kit with a Qiacube (QIAGEN), according to the manufacturer's instructions. RNA concentrations were assessed using a NanoDrop spectrophotometer (Thermo Fisher Scientific, Waltham, USA). Total RNA concentrations and available volumes of all samples are given in ESI Table S1.†

### Absolute quantification of miRNA by amplified TG-FRET

Absolute concentrations of unknown miRNAs were determined by using a calibration curve (FRET-ratio over concentration) constructed with the use of synthetic miRNAs with known concentrations (between 0.05 and 1.0 pM within 140 μl solutions). Unknown miRNAs were diluted at different dilution factors to be sure that their concentration range fitted the one of the calibration curves.

### RT-qPCR miRNA assays

TaqMan MicroRNA Assay Kits (Thermo Fisher) were used for all RT-qPCR experiments. All plasma RNA extracts were diluted five times. Breast cancer tissue RNA extracts were diluted to 0.5 ng μl^–1^ of total RNA, except for breast tumor samples 3, 4, and 6 (ESI Table S1†), which were diluted to 0.25 ng μl^–1^ of total RNAs. All other RNA extracts were diluted to 2 ng μl^–1^ of total RNA. RT reactions were carried out with a TaqMan MicroRNA Reverse Transcription Kit (Thermo Fisher) in 15 μl containing 5 μl of RNA extract, 0.15 μl of 100 mM dNTPs, 1 μl of Multiscribe reverse Transcriptase (50 U μl^–1^), 1.5 μl of 10× reverse transcription buffer, 0.19 μl of RNase inhibitor (20 U μl^–1^), 3 μl of gene-specific primer, and 4.16 μl of nuclease-free water. For synthesis of cDNA, the reaction mixtures were incubated at 16 °C for 30 min, at 42 °C for 30 min, at 85 °C for 5 min, and then held at 4 °C. Then, 1.33 μl of cDNA solution was amplified using 10 μl of TaqMan Universal PCR Master Mix II with UNG (Thermo Fisher), 1 μl of gene-specific primer and probe, and 7.67 μl of nuclease-free water in a final volume of 20 μl. Quantitative PCR was run on a PikoReal Real-Time PCR System (Thermo Fisher Scientific), and the reaction mixtures were incubated at 95 °C for 10 min, followed by 40 cycles of 95 °C for 15 s and 60 °C for 1 min. The quantitation cycles (*C*_q_) were calculated with PikoReal software (Thermo Scientific). Absolute concentrations of unknown miRNAs were determined by using a calibration curve (*C*_q_ over concentration) constructed with the use of synthetic miRNAs with known concentrations.

### Statistical analysis

#### TG-FRET

For statistical analysis and the estimation of LODs, all samples were prepared 3 times and measured in triplicates (*n* = 9) apart from the zero-concentration samples (without miRNA targets), which were prepared 10 times and measured in triplicates (*n* = 30). For specificity tests, all samples were prepared and measured once. For real sample detections, all samples were prepared in duplicate and measured once (*n* = 2). All RT-qPCR measurements were performed in duplicate and measured once (*n* = 2).

## Data availability

All source data of amplified TG-FRET and RT-qPCR measurements and all other relevant data are available from the corresponding author upon request.

## Conflicts of interest

The authors declare competing financial interests: N. H. and X. Q. are named inventors of a provisional European patent application number EP16305582.

## Funding

This work was supported by the European Commission (Innovative Medicine Initiative project “OncoTrack” and H2020-FET-Open project PROSEQO), the French National Research Agency (Investissements d’Avenir projects “Labex NanoSaclay: ANR-10-LABX-0035” and “IDEX Paris-Saclay” and ANR project “AMPLIFY”), the Institut Universitaire de France (IUF), the China Scholarship Council (CSC), the French “Institut National Du Cancer” and “Direction générale de l'offre de soins” (INCa and DGOS; project PRTk 16158 – Gynomir), the French “Institut national de la santé et de la recherche médicale” (INSERM), and the University of Montpellier.

## Author contributions

X. Q. designed the studies, performed sample preparation and experiments, analyzed the data, and wrote the manuscript. J. X. and J. G. performed sample preparation, RCA-FRET experiments, RT-qPCR experiments, and analyzed the data. A. Y.-A. performed RT-qPCR experiments. N. K. and P. B. prepared plasma samples and set up experimental conditions for optimal detection of miR-21 in those samples. C. R. and M. D. provided the RT-qPCR facility and guidance in its use and interpretation. C. U. and S. G. selected ovarian carcinoma donor patients and collected their informed consent. I. D.-R. and F. A. prepared THP-1 cells and samples, designed the THP-1 experiments, and analyzed the data. J. J. U. performed breast tissue sample preparation. N. G.-S. re-evaluated all used tumor tissues and collected tissue samples. J. H. diagnosed and re-evaluated all used tumor tissues and collected tissue samples. N. H. conceived and supervised the project, analyzed the data, and wrote the manuscript. All authors contributed in editing and writing of the manuscript and approved its final version.

## Supplementary Material

Supplementary information
