# Supplementary material for: Advanced microRNA-based cancer diagnostics using amplified time-gated FRET
Source: Chem Sci. 2018 Sep 11;9(42):8046–55. doi: 10.1039/c8sc03121e (PMC6249629; doi:10.1039/c8sc03121e)
Supplement: Supplementary file 1 [file SC-009-C8SC03121E-s001.pdf]

Electronic Supplementary Material (ESI) for Chemical Science.
This journal is © The Royal Society of Chemistry 2018

Advanced microRNA-based cancer diagnostics using amplified time-gated FRET

Xue Qiu, Jingyue Xu, Jiajia Guo, Akram Yahia-Ammar, Nikiforos-Ioannis Kapetanakis, Isabelle Duroux-Richard, Julia J. Unterluggauer, Nicole Golob-Schwarzl, Christophe Regeard, Catherine Uzan, Sébastien Gouy, Michael DuBow, Johannes Haybaeck, Florence Apparailly, Pierre Busson, Niko Hildebrandt*

ELECTRONIC SUPPORTING INFORMATION (ESI)

Figure S1. Absorption and emission spectra of the TG-FRET pairs………………………………..……….…….……….. 2

Figure S2. PL decays of the Tb-Cy5.5 FRET pair inside an RCP and inside a dsDNA…………...…………………….…. 3

Figure S3. Absolute concentrations of miR-21, miR-132, and miR-146a in the different samples……………………. 4

Figure S4. Value distributions and ROC curves ………………………………………………………………………….. 5

Table S1. Total RNA concentrations and sample volumes of clinical samples……………………………...…………….. 6

Table S2. Relevant information on human research participants………………………………………...……………… 7

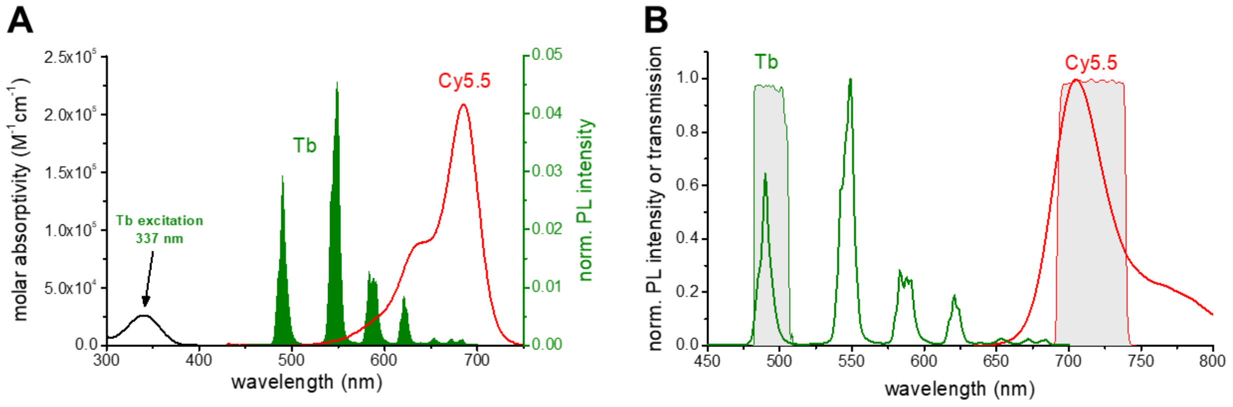

Figure S1. Absorption and emission spectra of the TG-FRET pair. **(A)** Spectra of Tb absorption (black) and emission (green) that partly overlaps with the absorption of Cy5.5 (red). Förster distance (the donor-acceptor distance for which FRET is 50 % efficient) was R_0 = 5.8 ± 0.2 nm and was calculated using $R_0 = 0.02108(\kappa^2 \Phi_D n^{-4} J)^{1/6}\ \mathrm{nm}$. The orientation factor κ^2 was taken as 2/3 because of random orientation of donor and acceptor during the FRET time (dynamic averaging), which is well justified by the long PL lifetime of the Tb donors and the unpolarized emission (fast isotropic rotation). The refractive index was *n* = 1.35 (aqueous buffer solution). The Tb-centered quantum yield was Φ_D = 0.80 ± 0.05. The overlap integral *J* was calculated by $J = \int_{475\,\mathrm{nm}}^{700\,\mathrm{nm}} I_D(\lambda)\varepsilon_A(\lambda)\lambda^4\,\mathrm{d}\lambda$ where $I_D(\lambda)$ is the emission intensity from the area-normalized (to unity) emission spectrum of Tb donor and $\varepsilon_A(\lambda)$ is the molar absorptivity of the acceptor. **(B)** Emission spectra of Tb (green) and Cy5.5 (red). Optical bandpass filter transmission spectra, which represent the detection channels for Tb and Cy5.5, are shown in gray.

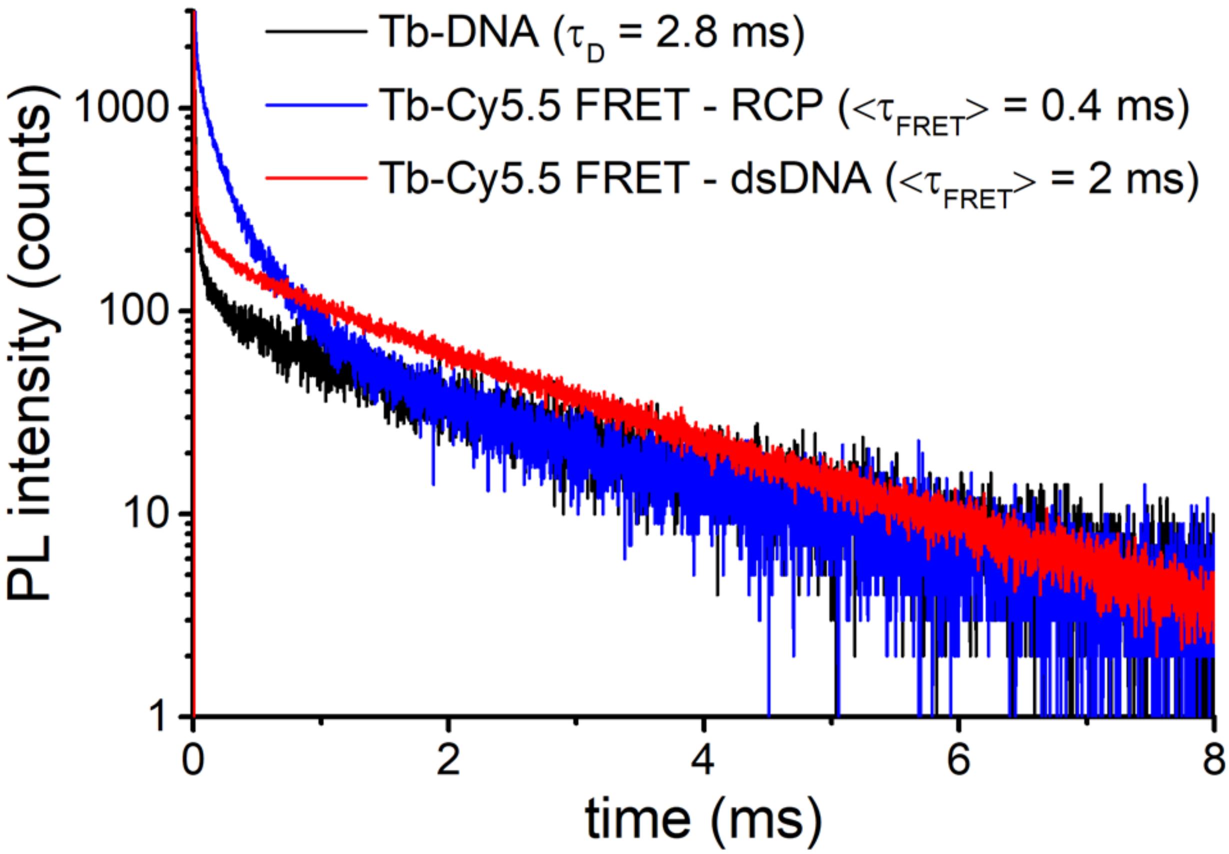

Figure S2. PL decays of Cy5.5 for a Tb-Cy5.5 donor acceptor pair at 18 base pairs distances in a dsDNA (red curve) and in a RCP concatemer (blue curve). A PL decay curve of Tb alone (black curve, no Cy5.5 present) is shown for comparison and for intensity normalization of the two PL decays (intensity normalized in the temporal region between 7 and 8 ms). The PL decay time of the donor in the absence of the acceptor (τ_{DA}) and of the acceptor in the presence of the donor (average FRET decay time <τ_{FRET}>) are given inside the graph. Resulting FRET-efficiencies ($E_{FRET} = 1 - <\tau_{FRET}>/\tau_D$) and Tb-Cy5.5 distances ($R = R_0 (<\tau_{FRET}>/(\tau_D - <\tau_{FRET}>))^{1/6}$) were E_{FRET}(RCP) = 0.86, E_{FRET}(dsDNA) = 0.28, R(RCP) = 4.3 nm, and R(dsDNA) = 6.8 nm. Förster distance was R_0 = 5.8±0.2 nm (*Small* **2017**, 13, 1700332). The distance calculation is based one a one donor / one acceptor FRET pair and does not take into account the coiled structure of the RCP, which may cause interaction of one donor with several acceptors.

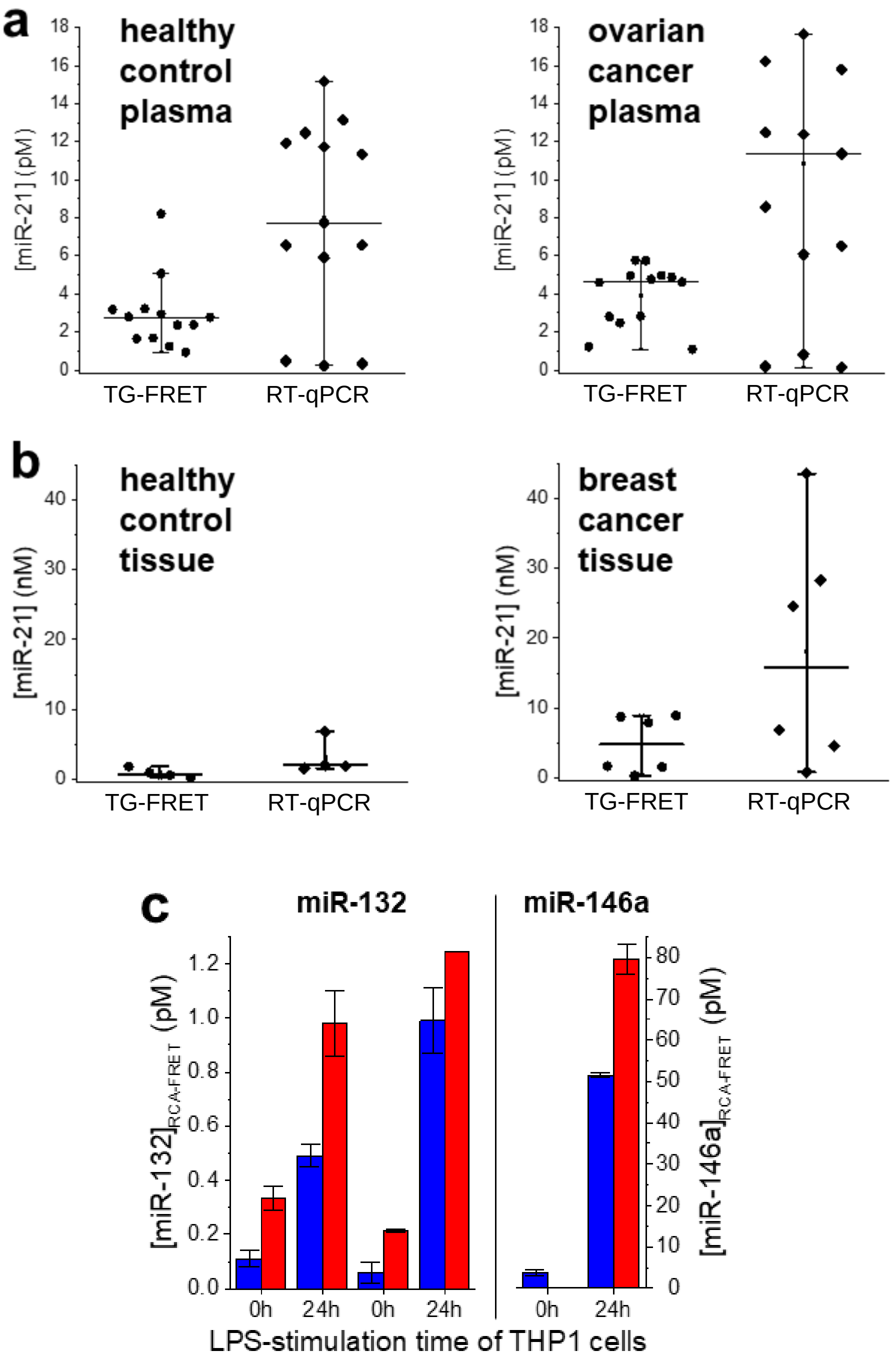

Figure S3. Absolute concentrations of miR-21, miR-132, and miR-146a in the different samples. Same data (but not normalized to the amount of total or small RNA) than in Figures 4 to 6 in the manuscript.

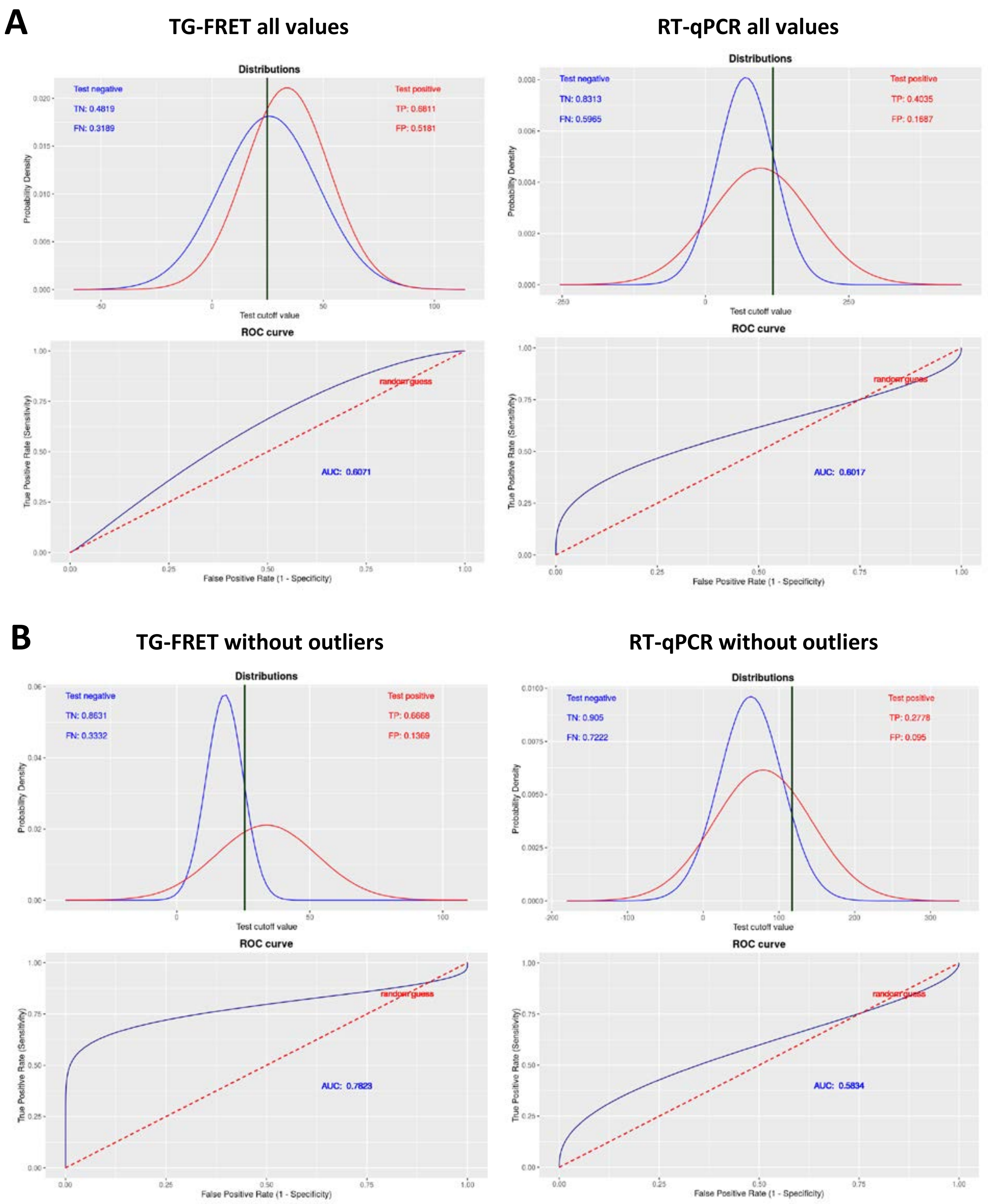

Figure S4. Distributions (top graphs – blue: healthy control plasma samples, red: ovarian cancer plasma samples) and receiver operating characteristic (ROC) curves (bottom graphs) for all values (**A**) and for the values without outliers (**B**) as shown in Figure 4 in the main manuscript. Curves were prepared using the ROC Curves Visual Tool (https://kennis-research.shinyapps.io/ROC-Curves/) that uses Shiny open source R package (https://www.rstudio.com/products/shiny/). All values are in amol/ng and multiplied by 100 to fit the value range of the ROC curve tool.

Table S1. Total RNA concentrations and sample volumes of clinical samples. Corresponding Figures in the manuscript are shown on the left hand column.

Figure	Extraction from	Sample name	Total RNA concentration in ng/µL	Available volume in µL
4a	Plasma	Control 1	14	50
	Plasma	Control 2	13	50
	Plasma	Control 3	10	50
	Plasma	Control 4	11	50
	Plasma	Control 5a (experiment 1)	12	50
	Plasma	Control 5b (experiment 2)	17	50
	Plasma	Control 6	13	50
	Plasma	Control 7	9	50
	Plasma	Control 8	11	50
	Plasma	Control 9	10	50
	Plasma	Control 10	11	50
	Plasma	Control 11	15	50
	Plasma	Control 12	19	50
	Plasma	Ovarian cancer 1	8	50
	Plasma	Ovarian cancer 2	15	50
	Plasma	Ovarian cancer 3	11	50
	Plasma	Ovarian cancer 4	13	50
	Plasma	Ovarian cancer 5	15	50
	Plasma	Ovarian cancer 6a (experiment 1)	10	50
	Plasma	Ovarian cancer 6b (experiment 2)	15	50
	Plasma	Ovarian cancer 7	11	50
	Plasma	Ovarian cancer 8	14	50
	Plasma	Ovarian cancer 9a (experiment 1)	12	50
	Plasma	Ovarian cancer 9b (experiment 2)	14	50
	Plasma	Ovarian cancer 10	12	50
	Plasma	Ovarian cancer 11	15	50
4b	Tissue	Control 1	133	26
	Tissue	Control 2	204	28
	Tissue	Control 3	160	28
	Tissue	Control 4	456	28
	Tissue	Breast tumor 1	1149	56
	Tissue	Breast tumor 2	44	28
	Tissue	Breast tumor 3	163	28
	Tissue	Breast tumor 4	1113	28
	Tissue	Breast tumor 5	916	28
	Tissue	Breast tumor 6	77	28
4c	Cells	0 h - 0.25 mio cells	203	30
	Cells	24 h - 0.25 mio cells	172	30
	Cells	0 h - 3 mio cells	180	180
	Cells	24 h - 3 moi cells	136	180

Table S2. Relevant information on human research participants.

**PLASMA SAMPLES**						
**Sample**	**Gender**	**Age (years)**	**Pathology**	**Tumor Stage**	**BRCA status**	**Treatment modalities**
13858	Female	52	None	None	WT	None
13993	Female	49	None	None	WT	None
14214	Female	42	None	None	WT	None
14168	Female	32	None	None	WT	None
14237	Female	52	None	None	WT	None
14267	Female	51	None	None	WT	None
14270	Female	25	None	None	WT	None
14394	Female	35	None	None	WT	None
14483	Female	56	None	None	WT	None
16400	Female	49	None	None	WT	None
17919	Female	43	None	None	WT	None
18189	Female	49	None	None	WT	None
Exo 015	Female	66	HGSOC	IIIc	WT	Neo-adjuvant chemotherapy, followed by debulking surgery and adjuvant chemotherapy
Exo 018	Female	55	HGSOC	IIIb	WT	Direct debulking surgery and adjuvant chemotherapy
Exo 025	Female	71	HGSOC	IIIc	WT	Neo-adjuvant chemotherapy, followed by debulking surgery and adjuvant chemotherapy
Exo 045	Female	66	HGSOC	Ia	WT	Direct debulking surgery and adjuvant chemotherapy
Exo 050	Female	63	HGSOC	IIIc	Unknown	Direct debulking surgery and adjuvant chemotherapy
Exo 069	Female	55	HGSOC	IIIc	Unknown	Neo-adjuvant chemotherapy, followed by debulking surgery and adjuvant chemotherapy
Exo 075	Female	58	HGSOC	IIIc	BRCA1 mut	Direct debulking surgery and adjuvant chemotherapy
Exo 079	Female	59	HGSOC	IIIc	Unknown	Neo-adjuvant chemotherapy, followed by debulking surgery and adjuvant chemotherapy
Exo 122	Female	61	HGSOC	IIIc	Unknown	Neo-adjuvant chemotherapy, followed by debulking surgery and adjuvant chemotherapy
Exo 129	Female	73	HGSOC	IIIc	WT	Neo-adjuvant chemotherapy, followed by debulking surgery and adjuvant chemotherapy
Exo 141	Female	39	HGSOC	IIIc	WT	Chemotherapy alone

**TISSUE SAMPLES**						
**Sample**	**Gender**	**Age (years)**	**Pathology**	**Tumor Staging**	**receptor status**	**Treatment modalities**
nnB1	Female	55	material far away from nB1 tumor	/	/	see nB1
nnB2	Female	78	no history of breast cancer	/	/	no breast cancer-specific treatment
nnB3	Female	80	no history of breast cancer	/	/	no breast cancer-specific treatment
nnB4	Female	70	history of breast cancer for the not sampled breast (more than 10 years ago)	/	/	no breast cancer-specific treatment for more than ten years
nB1	Female	55	poorly differentiated invasive breast cancer without special subtype, metastasising	apT-4b, G-3, V-1, L-1, pM-1	estrogen receptor: moderately positive, progesterone receptor: moderately positive, no Her2 overexpression	no surgery, no breast cancer-specific treatment
nB2	Female	49	highly differentiated invasive ductal breast cancer	G1 pT1c(m) N1mi (1/3;sn)	estrogen receptor: positive, progesterone receptor: positive, no Her2 overexpression	direct surgery (sampling), post operative radiation therapy, adjuvant anti-hormone therapy
nB3	Female	50	moderately differentiated invasive ductal breast cancer	pT1c, G2, pN0(sn, 0/1) pTis(DCIS), G2	estrogen receptor: positive, progesterone receptor: positive, no Her2 overexpression	direct surgery (sampling), post operative radiation therapy, adjuvant anti-hormone therapy
nB4	Female	65	poorly differentiated invasive ductal breast cancer, metastasising	G3 pT4b L1	estrogen receptor: positive, progesterone receptor: negative, no Her2 overexpression	direct surgery (sampling), post operative radiation therapy, palliative adjuvant anti-hormone therapy
nB5	Female	47	moderately differentiated invasive ductal breast cancer	G-2, pT-X	estrogen receptor: positive, progesterone receptor: positive, no Her2 overexpression	direct surgery (sampling), post operative radiation therapy, adjuvant chemotherapy, adjuvant anti-
nB6	Female	77	moderately differentiated invasive ductal breast cancer	G2 pT2	estrogen receptor: positive, progesterone receptor: moderately positive, no Her2 overexpression	direct surgery (sampling), post operative radiation therapy, adjuvant anti-hormone therapy
